# A Case Report on a Unique Explanation for Headache With Ophthalmoplegia: The Tolosa-Hunt Syndrome

**DOI:** 10.7759/cureus.26093

**Published:** 2022-06-19

**Authors:** Ayat Alhakeem, Moustafa M Elziny, Mansour Elmahdi

**Affiliations:** 1 Department of Family Medicine, Primary Health Care Corporation, Doha, QAT; 2 Department of Academic Internal Medicine and Geriatrics, University of Illinois at Chicago, Chicago, USA

**Keywords:** ptosis, eye pain, ophthalmoplegia, headache, tolosa-hunt syndrome

## Abstract

Tolosa-Hunt syndrome (THS) is an extremely rare disorder characterized by painful unilateral ophthalmoplegia triggered by idiopathic inflammation of the cavernous sinus affecting the third, fourth, and/or sixth cranial nerve. Corticosteroid therapy effectively improves THS symptoms; thus, early clinical suspicion and diagnosis are essential. We report the case of a 37-year-old patient who presented with left-sided eye pain and double vision for four days. Physical examination was significant for oculomotor, trochlear and abducent nerves palsies on the left eye with slow light reflex. Contrast-enhanced magnetic resonance imaging of the head displayed mild asymmetry of the cavernous sinus regions with fullness on the left side and focal lateral thickening, confirming the THS diagnosis. The patient’s symptoms improved dramatically upon starting oral corticosteroid therapy.

## Introduction

Tolosa-Hunt syndrome (THS) is an extremely rare disorder with an estimated annual incidence of one case per million per year [1]. THS is characterized by painful unilateral ophthalmoplegia triggered by idiopathic inflammation of the cavernous sinus. THS was first described in 1954, and its marked responsiveness to glucocorticoid treatment was described a few years later [2,3]. THS has a similar incidence between men and women and can occur in any age group [4].

Although considered a benign condition that typically resolves spontaneously within several weeks [3], residual cranial nerve palsies are possible, and relapses are frequent, often necessitating extended immunosuppressant therapy.

Patients present with a constant gnawing pain behind the eye that may begin several days prior to ophthalmoplegia. Patients can also present with dysfunction of one or more of the cranial nerves III, IV, VI, and the cranial nerve V ophthalmic branch due to the inflammation within the cavernous sinus. Though classically unilateral, bilateral symptoms occur in 4-5% of cases [5].

The diagnosis of Tolosa-Hunt syndrome is suggested by the clinical manifestations, supported by neuroimaging results and the clinical response to corticosteroids.

The THS diagnostic criteria recommended by the International Headache Society include a unilateral headache, in addition to Granulomatous inflammation of the cavernous sinus, superior orbital fissure or orbit evidenced by magnetic resonance imaging (MRI) or biopsy, along with weakness of one or more of the ipsilateral III, IV, and/or VI cranial nerves. The headache must be ipsilateral to the inflammation site and develops with or precedes the oculomotor paresis by ≤2 weeks, and an alternative diagnosis does not better account for symptoms [6].

A contrast-enhanced MRI is crucial to the diagnostic workup of a patient with painful ophthalmoplegia, chiefly to exclude other causes [5,7]. In patients with THS, MRI features can include enlargement of the cavernous sinus with abnormal tissue that is strongly enhanced with gadolinium, abnormal convexity of the wall of the cavernous sinus, and focal narrowing of the intra-cavernous internal carotid artery [8-10].

Glucocorticoid administration has diagnostic and therapeutic values. Rapid pain resolution (within 24 to 72 hours) confirms suspected THS [4,5,11]. And Improvement of cranial nerve palsies with regression of MRI abnormalities over the next two to eight weeks provides added confirmation of the diagnosis [7].

## Case presentation

We present the case of a male 37-year-old patient from an Asian background, known to have primary hypertension, who presented to the clinic with the complaint of left-sided eye pain and double vision for four days, without any associated fever, headache, vomiting, limb weakness/numbness or trauma.

During a physical exam, his vital signs, including blood pressure, were within normal range. The neurological exam showed intact motor and sensory functions. The cranial nerve and eye exam revealed complete ophthalmoplegia of the left eye, with sluggish light reflex. The right eye exam was normal, and there was no other focal motor or sensory neurological deficit.

The patient was transferred immediately to the emergency department (ED) for further assessment and management. Upon ophthalmologic and neurologic examination at the ED, he was found to have a frozen left globe, indicating oculomotor, trochlear and abducent nerves palsies on the left eye, with mild ptosis and slow light reflex.

Complete blood count, inflammatory markers, and full biochemistry tests, including thyroid and liver function tests, were within the normal range. Chest X-ray and computed tomography head scan were also normal. Lumbar puncture revealed normal cerebrospinal fluid analysis, excluding brain malignant and infectious causes.

**Figure 1:**
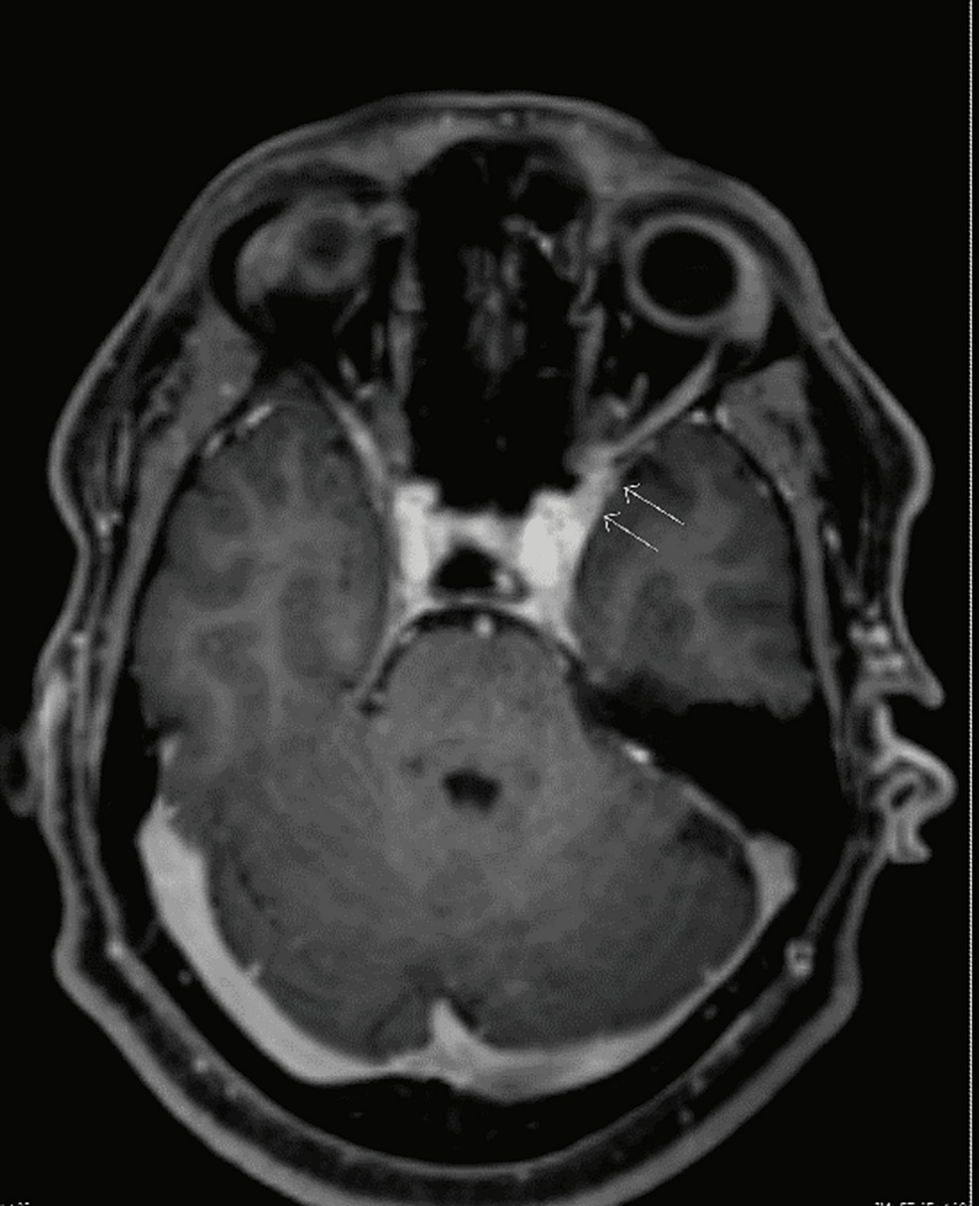
MRI of the brain in T1 view with contrast enhancement showing mild asymmetry of the cavernous sinus regions with relative fullness on the left side and focal lateral thickening. MRI = magnetic resonance imaging

Contrast-enhanced magnetic resonance imaging of the head displayed mild Asymmetry of the cavernous sinus regions with relative fullness on the left side and focal lateral thickening, which was more prominent post-contrast enhancement encroaching the orbital apex and superior orbital fissures (Figure 1). Without any evidence of intracranial hemorrhage or venous sinus thrombosis.

Accordingly, the patient was admitted and commenced on daily oral 60 mg of prednisolone for two weeks with a plan to taper it down. Symptoms subsequently improved within 72 hours, and the patient was discharged home and given an outpatient follow-up appointment.

## Discussion

When evaluating patients with a headache in primary care or the emergency department, the most crucial task is to confirm the absence of alarming symptoms, as these can point to a severe secondary cause [12]. One of the less-known causes of craniofacial pain is the Tolosa-Hunt syndrome (THS), which consists of eye pain and ocular muscle weakness attributed to one or multiple ocular motor cranial nerve palsies. Various blends of these cranial nerve palsies may occur, localizing the pathological process to the region of the cavernous sinus/superior orbital fissure [13].

Although it is known to be a benign condition, patients need to be promptly assessed to exclude tumors, vascular and other inflammatory pathologies and for early treatment once the diagnosis is confirmed. Even after treatment, half of the patients with THS will experience recurrence within the coming months or years. Recurrence can happen at the same site, contralateral site or occasionally bilateral [13].

Since the differential diagnosis is broad, and patients can easily get misdiagnosed, a confirmed diagnosis of THS can only be reached after excluding other mimicking entities. Cavernous sinus thrombosis is one of the chief differential diagnoses; it can be septic, secondary to an infectious process, or aseptic. The patient can present with proptosis, lid swelling, lacrimation and chemosis in conjunction with eye pain and ophthalmoplegia [13].

Magnetic resonance imaging (MRI) is the most valuable imaging modality to differentiate THS from other differential diagnoses. It offers accurate assessment and facilitates further plans of management [7]. However, cases with THS without any MRI features have been reported in the literature [14].

A recently conducted study in Qatar described the demographics of 31 patients (representing all those diagnosed with THS between January 2015 and December 2020) and mentioned that 71% of patients were males and 29% were females. The median age was 40 years. Most patients presented with disturbed vision; 70.9% of those had third nerve palsy. Abnormal MRI findings were detected in 64.5%. Most patients received steroids, with a response rate of 70.9% and a recurrence rate of 9.7%. Previous episodes of THS and the female gender were linked with a higher recurrence rate (p-value 0.009 and 0.018) [15].

## Conclusions

Tolosa Hunt syndrome (THS) is considered one of the rare conditions documented by the National Organization for Rare Disorders (NORD), and very little is known about it at the primary care level. Patients with THS present with painful ophthalmoplegia with a dramatic response to systemic corticosteroids. Given the significance of improved coordination amongst the interprofessional team at the primary and secondary level and for better delivery of care for patients affected by this syndrome, clinicians need to be aware of the different causes of painful ophthalmoplegia, including the less common presentations.
